# Integrating DynamiCROP model and risk assessment for pesticide residues in spinach: Implications for food safety

**DOI:** 10.1002/ps.70799

**Published:** 2026-04-12

**Authors:** Ji‐Woo Yu, Min‐Ho Song, Jung‐Hoon Lee, Hui‐Yeon Ahn, Eun‐Song Choi, Young‐Soo Keum, Hyun Ho Noh, Ji‐Ho Lee

**Affiliations:** ^1^ Department of Crop Science, College of Sang‐Huh Life Science Konkuk University Seoul Republic of Korea; ^2^ School of Natural Resources and Environment Science, College of Agriculture and Life Sciences Kangwon National University Chuncheon Republic of Korea; ^3^ Department of Food Biotechnology and Environmental Science Kangwon National University Chuncheon Republic of Korea; ^4^ Residual Agrochemical Assessment Division National Institute of Agricultural Sciences Wanju Republic of Korea

**Keywords:** DynamiCrop, pesticide, spinach, predictive model, risk assessment, food safety

## Abstract

**BACKGROUND:**

Reliable prediction of pesticide residues in leafy vegetables is essential for dietary risk assessment and regulatory decision‐making. This study aimed to crop‐specifically parameterize the dynamiCROP model for spinach, a species not included in the default database, to predict pesticide residue dynamics and assess associated dietary risks.

**RESULTS:**

Nine commonly used pesticides were applied under controlled field conditions, and their dissipation patterns were evaluated using both experimental measurements and model simulations. Spinach‐specific parameters, including leaf area index and growth stages, were incorporated to improve predictive accuracy. The model showed strong performance, with R^2^ values exceeding 0.8 and mean absolute error (MAE) values remaining below the corresponding maximum residue limits (MRLs). Risk assessments based on both measured and simulated data indicated that propamocarb exhibited the highest hazard quotient (HQ), although all pesticides showed HQ values below 10% 14 days after application.

**CONCLUSION:**

The results demonstrate that dynamiCROP reliably predicts pesticide residue dynamics in spinach and supports dietary risk assessment. This study extends the applicability of the model to leafy vegetables, which are widely consumed globally, addressing a key gap in pesticide residue modeling and supporting science‐based regulatory management. © 2026 The Author(s). *Pest Management Science* published by John Wiley & Sons Ltd on behalf of Society of Chemical Industry.

## Graphical abstract

This study adapted and validated the dynamiCROP model for spinach, a leafy vegetable not previously included in its standard framework. The findings confirm that model‐predicted pesticide concentrations, when integrated into hazard quotient (HQ) calculations, can effectively support dietary risk assessment.

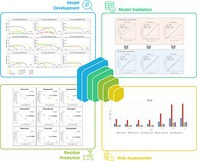

## 1 INTRODUCTION

Spinach (*Spinacia oleracea L*.) is a globally significant leafy vegetable, widely consumed for its high nutritional value, including vitamins, minerals, and bioactive compounds such as flavonoids and carotenoids.^1^, ^2^ With an annual global production exceeding 30 million tons, spinach is a dietary staple, particularly in Asia, North America, and Europe, where its consumption is integral to various cuisines.^3^, ^4^ However, due to its large surface area and high water content, spinach has frequently been associated with pesticide residues. According to the Environmental Working Group's 2024 ‘Dirty Dozen’ report, spinach ranks as the second most pesticide‐contaminated crop, highlighting the urgent need for research into pesticide dynamics and their implications for consumer health.^4^, ^5^

The use of pesticides is critical in ensuring the yield and quality of spinach crops by protecting them from pests and diseases.^6^, ^7^ However, their application raises significant concerns regarding the accumulation of pesticide residues in edible parts, which can pose health risks to consumers and contribute to environmental pollution.^8^, ^9^ To address these challenges, an accurate assessment of pesticide residue patterns in crops is essential.^10^ These assessments can be foundational for establishing regulatory limits, guiding safe application practices, and ensuring sustainable agricultural systems.^11^

The dynamiCROP model has emerged as a valuable tool for modeling pesticide behavior in crops. Developed by Peter Fantke and collaborators at the Swiss Federal Institute of Technology (ETH Zurich) and later refined by international teams, dynamiCROP is designed to simulate the uptake, translocation, dissipation, and residue behavior of pesticides in crops.^12^, ^13^ The model integrates plant‐specific, chemical‐specific, and environmental parameters to predict pesticide dynamics across different compartments, including soil, air, leaves, and roots.^14^ DynamiCROP has been applied extensively in crops such as wheat, tomatoes, and cucumbers to evaluate pesticide residues and support dietary risk assessments.^15^ Its ability to account for multiple environmental and agronomic factors makes it a versatile tool in agricultural research and regulatory decision‐making.^16^

However, spinach is not included in dynamiCROP's default database despite its global consumption. This study customized the model for spinach by optimizing key plant‐specific parameters, including leaf area index, water and lipid content, growth stages, and crop density. This optimization ensured that the unique characteristics of spinach were accurately represented in the simulations, enabling precise modeling of pesticide dynamics.^17^ By tailoring the dynamiCROP model to spinach, this research extends its application to a new crop, enhancing its utility in pesticide risk assessment.

This study aimed to analyze the behavior of nine commonly used pesticides in spinach using the optimized dynamiCROP model. Although the dynamiCROP model has been used across a variety of crops, it lacks specific parameterization for spinach, which is highly consumed and has high pesticide accumulation. This research addresses that limitation by introducing crop‐specific physiological and phenological adjustments. Through field trials and model simulations, it investigated the uptake, distribution, and dissipation of these pesticides, evaluated residue levels at harvest, and assessed dietary risks associated with spinach consumption. While not developing a novel algorithm, this work fills an important application gap by localizing the dynamiCROP model to a high‐risk, high‐consumption leafy crop such as spinach. Integrating experimental data with advanced modeling provides a robust framework for understanding pesticide dynamics in spinach cultivation, contributing to improved pesticide management, enhanced food safety, and the development of evidence‐based regulatory policies.

## 2 MATERIALS AND METHODS

### 2.1 Chemicals and reagents

Pesticide standards of pencycuron (99.8%), propamocarb (99.7%), deltamethrin (99.7%), cypermethrin (95.2%), thiamethoxam (99.7%), fenazaquin (99.1%), dinotefuran (98.0%), cymoxanil (99.9%), and fenpropathrin (98.0%) were purchased from Sigma‐Aldrich (St. Louis, MO, USA). The detailed physicochemical properties of each compound are described in Table 3. Solvents, including high‐performance liquid chromatography (HPLC)‐grade acetonitrile, water, and hexane, were supplied by J.T. Baker (Phillipsburg, NJ, USA). Additional reagents such as ammonium formate (≥ 99.0%) and formic acid (≥ 98.0%) were also obtained from Sigma‐Aldrich.

Sample extraction and cleanup were performed using QuEChERS (Quick, Easy, Cheap, Effective, Rugged, and Safe) extraction kits (EN 15662, 4 g MgSO_4_, 1 g NaCl, 1 g Na‐Citrate, 0.5 g Disodium Citrate Sesquihydrate) and QuEChERS dispersive solid‐phase extraction (d‐SPE) kits (EN 15662, 150 mg MgSO_4_, 25 mg Primary Secondary Amine, PSA) purchased from CTK Corporation (Daejeon, Republic of Korea). Florisil cartridges for solid‐phase extraction (SPE) were also purchased from CTK Corporation. All chemicals and reagents used in this study were analytical or HPLC grade, ensuring reliability and reproducibility in pesticide residue analysis.

### 2.2 Field trials

The greenhouse experiment was designed to represent a worst‐case scenario, in which environmental conditions favor higher pesticide residues, ensuring a conservative evaluation of dietary exposure. The greenhouse was located in Geumnam‐ri, Waegwan‐eup, Chilgok‐gun, Gyeongsangbuk‐do, Republic of Korea, from 2016 to 2022 (Table S1). The greenhouse environment was maintained with temperature and humidity ranges presented in Table S2. All field treatments were conducted in triplicate plots. Considering the usage frequency in Korean spinach production and the range of chemical classes, nine pesticides were selected (Table S3). They were applied during the experiment, comprising three fungicides, which were sprayed three times, and six insecticides, which were sprayed twice. Following pesticide application, more than 1 kg of spinach was harvested from each treatment plot.

Pesticides were applied at a dosage of 200 L per 10 a using foliar spraying methods. The commercial pesticide formulations used in this study were purchased from a Korean agrochemical company: Kyungnong, Seoul, for pencycuron, cypermethrin, Dongbangagro, Seoul, for propamocarb, Bayer CropScience Korea, Seoul, for deltamethrin, Syngenta Korea, Seoul, for thiamethoxam and cymoxanil, Farmhannong, Seoul, for fenazquin, Nonghyup Chemical, Seongnam for dinotefuran, and fenpropathrin. Applications were conducted with an electric sprayer, ensuring thorough coverage until the spray solution visibly dripped from the leaves. The spinach planting density was maintained at 10 cm × 10 cm, providing uniform growth conditions across all plots.

### 2.3 Pesticide extraction methods from spinach

For pencycuron, 20 g of homogenized spinach sample was mixed with 100 mL of acetone and ground using a homogenizer for 3 min. The mixture was shaken for 1 h and filtered through a Büchner funnel under vacuum. The residue was washed with 30 mL of acetone, and the filtrate was evaporated under reduced pressure. The concentrate was transferred to a 250 mL separatory funnel and sequentially mixed with 50 mL of saturated saline solution and 50 mL of distilled water. The sample was then partitioned with 100 and 50 mL of hexane, respectively. The hexane layers were combined, passed through 50 g of sodium sulfate for dehydration, and evaporated under reduced pressure. The dried extract was reconstituted in 10 mL of hexane for further purification. Solid‐phase extraction (SPE) was performed using a Florisil 1000 mg cartridges (1000 mg, 6 mL; CTK Corporation, Daejeon, Korea) were used for sample cleanup. cartridge preconditioned with 10 mL of hexane. A 2 mL aliquot of the sample was loaded onto the cartridge and eluted sequentially with 30 mL of hexane (discarded) and 30 mL of ethyl acetate/hexane (20/80, v/v). The second eluate was evaporated under reduced pressure, reconstituted in 5 mL of acetonitrile, and analyzed using high‐performance liquid chromatography (HPLC).

For propamocarb, 20 g of spinach sample was mixed with 100 mL of acetone/0.1 M HCl (hydrogen chloride) (70/30, v/v) and homogenized for extraction. The extract was filtered through a Büchner funnel, and the acetone in the filtrate was evaporated. The aqueous phase was partitioned twice with 50 mL of diethyl ether, and the diethyl ether layers were discarded. The remaining aqueous layer was treated with 1 g of sodium carbonate and partitioned twice with 50 mL of diethyl ether. The combined ether extracts were evaporated under reduced pressure, reconstituted in 4 mL of ethyl acetate, and analyzed using gas chromatography with a nitrogen‐phosphorus detector (GC‐NPD).

For cypermethrin and deltamethrin, 10 g of spinach sample was extracted with 100 mL of acetone and filtered through a Büchner funnel under vacuum. The filtrate was transferred to a 1 L separatory funnel containing 50 mL of saturated saline solution and 450 mL of distilled water. The sample was partitioned with 200 and 150 mL of hexane, respectively. The hexane layers were combined, evaporated under reduced pressure, and reconstituted in 10 mL of hexane for purification. Purification was performed using an SPE Florisil 1000 mg cartridge preconditioned with 10 mL of hexane. A 4 mL aliquot of the sample was loaded, and the cartridge was eluted with 30 mL of dichloromethane. The eluate was evaporated under reduced pressure, reconstituted in 4 mL of acetone, and analyzed using gas chromatography with an electron capture detector (GC‐ECD).

For the remaining pesticides (thiamethoxam, fenazaquin, dinotefuran, cymoxanil, and fenpropathrin), residues were extracted and purified using the QuEChERS method (EN 15662). Briefly, 10 g of homogenized spinach sample was mixed with 10 mL of acetonitrile and processed with a QuEChERS extraction kit. The extract was cleaned using a dispersive solid‐phase extraction (d‐SPE) kit and analyzed using liquid chromatography–tandem mass spectrometry (LC–MS/MS) and gas chromatography‐tandem mass spectrometry (GC–MS/MS) for fenpropathrin. Detailed instrumental conditions for pesticide quantification are provided in Table S4.

### 2.4 Pesticide residue and dissipation kinetics

Pesticide residues were extracted and analyzed using different methods depending on the chemical properties of the compounds. The total pesticide residue in spinach was quantified based on the measured concentrations in the edible parts. The dissipation of pesticide residues over time was modeled using first‐order kinetics. The dissipation rate constant (*k*) and half‐life (*t*_*1/2*_) were calculated using the following equations:

$$C_{t}=C_{0}\times e^{-\mathrm{kt}}$$

$$t_{1/2}=\frac{\ln\left(2\right)}{k}$$

where $C_{t}$: pesticide concentration at time t (mg/kg). $C_{0}$: initial pesticide concentration at time t = 0 (mg/kg). *k*: dissipation rate constant (day^−1^). t: time after pesticide application (days)

The dissipation rate constant (k) was estimated by fitting the measured residue data to the exponential decay equation using non‐linear regression analysis. The half‐life (*t*_*1/2*_) of each pesticide was then calculated to evaluate the persistence of residues in spinach.

All calculations were performed using statistical software to ensure precision and reproducibility. These parameters were used to assess the behavior of each pesticide in spinach and provide insights into their potential environmental and health impacts.

### 2.5 Optimization and validation of the model

The behavior and dissipation of pesticides in spinach were simulated using the dynamiCROP model frame, a dynamic plant uptake and pesticide residue prediction tool. Since spinach‐specific parameters are not available in the default model database, the simulation was conducted using parameters from lettuce as a base crop, with modifications tailored to spinach (Table 1). The residual concentration after multiple applications was calculated using the following equation:

$$C_{\left(\text{final}\right)}=C_{1}+C_{2}+\cdots +C_{n}$$

where *n* is the total number of applications, and $C_{n}$ is the modeled concentration after the *n*th application.

**Table 1:** Crop‐specific parameters for spinach input in DynamiCrop

Crop specific parameter	Unit	Default model plant data (lettuce)	Spinach	Reference
Organic carbon content in soil	kg/kg	0.02	0.02	23
pH in (agricultural) soil	‐	7.0	4.7	23
Depth of soil	m	0.3	0.5	23
Yield (fresh weight) of harvested plant (agricultural output)	kg/m^2^	3.33	2.65	Experimental value
Ratio between root and aerial plant parts	kg_root_/kg_plant,aerial_	0.09	0.09	24
Fraction of the aerial plant parts that is stem	kg_stem_/kg_plant,aerial_	0.12	0.15	24
Fraction of the aerial plant parts that is leaf	kg_leaf_/kg_plant,aerial_	0.88	0.85	24
Leaf area index when plant starts to grow (lettuce: leaf cover [m^2^])	m^2^_leaf_/m^2^_surface_	0.03	0.03	24
Leaf area index when plant is harvested	m^2^_leaf_/m^2^_surface_	n/a	2.6	24
Leaf area index when substance is applied	m^2^_leaf_/m^2^_surface_	n/a	2.6	24
Leaf cover when plant is harvested	m^2^	n/a	2.6	24

Actual experimental data, including weather conditions (*e.g*., temperature, humidity, and rainfall) and pesticide application details (*e.g*., dosage, application frequency, and method), were incorporated into the model to ensure realistic simulations. Crop‐specific parameters, such as leaf area index, growth stages, and water and lipid content, were derived from a thorough review of the scientific literature and adjusted to reflect the unique physiological characteristics of spinach. These adjustments allowed the model to simulate pesticide behavior more accurately in spinach under the tested conditions.

The dynamiCROP model was employed to simulate the uptake, translocation, dissipation, and residue levels of the nine pesticides applied in this study. The simulation results were compared with measured residue data to evaluate the model's predictive accuracy and assess its applicability to spinach cultivation.

### 2.6 Statistical analysis

The model's performance was evaluated by comparing the measured pesticide residue data with model‐predicted values using multiple statistical parameters. The coefficient of determination (*R* ^*2*^) was calculated using the formula:

$$R^{2}=1-\frac{\mathrm{SSE}}{\mathrm{SST}}$$

where SSE is the sum of squared errors ($\sum {\left(A_{i}-F_{i}\right)}^{2}$), SST is the total sum of squares ($\sum {\left(A_{i}-\bar{A}\right)}^{2}$), with $A_{i}$ representing the measured pesticide residue concentration (mg/kg), $F_{i}$ the model‐predicted concentration, and $\bar{A}$ the arithmetic mean of measured concentrations across all sampling times. This formulation ensures that $R^{2}$ reflects the proportion of the variance in the measured data explained by the model.^16^

To evaluate the predictive accuracy of the model, the Mean Absolute Error (MAE) was calculated using the following equation:

$$\mathrm{MAE}=\frac{1}{n}\times \sum_{i=1}^{n}|A_{i}-F_{i}|$$

MAE quantifies the average absolute difference between measured and predicted values, providing an intuitive measure of the model's accuracy. Lower MAE values indicate better agreement between the model predictions and experimental data.

The relative root mean square error (RRMSE) was also calculated to evaluate the accuracy of the model. RRMSE was determined using the equation:

$$\text{RRMSE}=\sqrt{\frac{\sum_{i=1}^{n}{\left(A_{i}-F_{i}\right)}^{2}}{n\times \left(\sum_{i=1}^{n}F_{i}^{2}\right)}\times 100\%}$$

This metric quantifies the relative error between measured and predicted values, with lower RRMSE values indicating better model performance.

Additionally, the root mean squared logarithmic error (RMSLE) was calculated to assess the model's ability to predict values across a wide range of data scales. RMSLE was computed as:

$$\text{RMSLE}=\sqrt{\frac{1}{n}\sum_{i=1}^{n}{\left(\log\left(1+A_{i}\right)-\log\left(1+F_{i}\right)\right)}^{2}}$$

RMSLE is particularly effective for datasets with large variations, as it minimizes the impact of extreme values.

Statistical analyses were conducted using both measured and simulated data, allowing for a comprehensive evaluation of the model's predictive accuracy. The results were used to assess the model's suitability for simulating pesticide residue dynamics in spinach and its potential applicability in dietary risk assessments.

### 2.7 Risk assessment

The potential health risks associated with pesticide residues in spinach were evaluated by calculating the hazard quotient (HQ) using both measured and model‐simulated data. The HQ was determined using the formula:

$$\mathrm{HQ}=\frac{\text{Estimated Daily Intake}\;\left(\mathrm{EDI}\right)}{\text{Acceptable Daily Intake}\;\left(\mathrm{ADI}\right)}\times 100$$

The estimated daily intake (EDI) was calculated as follows:

$$\mathrm{EDI}=\text{Residual Concentration}\;\left(\frac{\mathrm{mg}}{\mathrm{kg}}\right)\times \text{Daily Food Intake}\;\left(\frac{g}{\mathrm{Day}}\right)$$

Spinach residue concentrations were obtained from both field‐measured data and model predictions. The average daily spinach consumption and the standard body weight were based on dietary surveys relevant to the study population. For the acceptable daily intake (ADI) values, the lowest value established by regulatory authorities from Korea, Codex, Japan, the United States, and the European Union was selected to ensure a conservative and robust risk assessment.

## 3 RESULTS AND DISCUSSION

### 3.1 Measured residual pesticide

The analytical methods for nine pesticides were optimized in terms of linearity, accuracy, and precision (Table S5). The matrix‐matched calibration curves showed reliable linearity, with the R^2^ ranging from 0.9953–1.0000 (Table S5). For validated accuracy and precision, recovery tests were performed at two or three levels, including LOQ (limit of quantitation). The recovery rate ranged from 70.4% to 118.7%, and the relative standard deviation was between 0.8% and 15.9%. These recovery results confirmed that the analytical methods were suitable for quantitation of nine pesticides.

**Figure 1:**
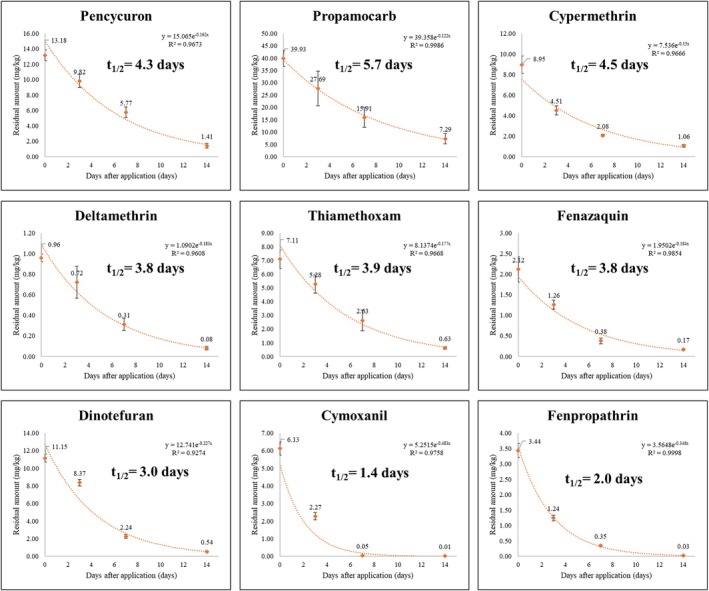
Dissipation patterns and half‐life of nine pesticides in spinach.

The dissipation patterns of nine pesticides applied to spinach were analyzed to determine their residual levels over time. Since these chemicals had various stability, solubility and half‐lives, the residual amounts were different (Table 2). Figure 1 and Table S6 present the degradation trends of these pesticides, revealing significant differences in their dissipation rates. Nine pesticides were degraded in spinach according to a first‐order equation. All pesticides degraded with time, exhibiting an R^2^ value above 0.9 (Fig. 1). Residual amounts of pesticides ranged between 0.01 and 43.68 mg/kg. Among the nine pesticides, the highest maximum average residual concentration was observed for propamocarb, reaching 39.39 mg/kg. A previous study revealed that propamocarb residues in small radish leaves ranged from 24 to 42 mg/kg, with an outlier recorded at 267 mg/kg.^18^ Because of this high concentration residual pattern, EFSA suggested that the MRL for small radish leaves should increase from 30 to 600 mg/kg.^18^ The high residue levels of propamocarb in leafy vegetables are attributed to its high water solubility (>9 × 10^5^ at pH 7.0), low volatility (1.5 × 10^−4^ Pa m^2^ mol^−1^, calc.), and metabolic stability (DT_50lab, 20°C_ = 10.9 ~ 136 days), which allow it to persist on leaf surfaces with minimal degradation (Table S3).^19^, ^20^ This indicates that propamocarb exhibits high initial residue in spinach, likely due to its physicochemical properties, including solubility and adsorption characteristics.

**Table 2:** Physicochemical properties and maximal residual limits (MRL) of nine pesticides

Pesticide	Molecular weight (g/mol)	Kow	MRL (mg/kg)	t_1/2, spinach_ (days)	t_1/2, soil_ (days)	m_appilied_ (kg/m^2^)
Pencycuron^a^	328.84	4.9 × 10^3^	20 (leafy vegetable)	4.3	64	2.0 × 10^−4^
Propamocarb^a^	188.30	6.0 × 10^3^	25 (leafy vegetable)	5.7	35.4	1.3 × 10^−4^
Cypermethrin^b^	416.30	8.6 × 10^4^	5 (spinach)	4.5	31	1.0 × 10^−4^
Deltamethrin^b^	505.20	1.0 × 10^7^	0.05 (spinach)	3.8	24	2.0 × 10^−6^
Thiamethoxam^b^	291.71	7.0 × 10	5.0 (leafy vegetable)	3.9	39	1.0 × 10^−5^
Fenazaquin^b^	306.40	2.6 × 10^4^	0.7 (leafy vegetable)	3.8	30.5	6.7 × 10^−6^
Dinotefuran^b^	202.21	2.6 × 10	10 (spinach)	3.0	82	1.0 × 10^−5^
Cymoxanil^a^	198.18	4.4 × 10	19 (spinach)	1.4	25.3	2.1 × 10^−5^
Fenpropathrin^b^	349.42	5.0 × 10^3^	5.0 (spinach)	2.0	28	1.0 × 10^−5^

^a^ Fungicide.

^b^ Insecticide.

Cymoxanil demonstrated the shortest half‐life (1.4 days), indicating its rapid degradation in the spinach environment. Previous studies indicated that cymoxanil had rapidly degraded in plants: 0.5–0.7 days in grapes and 2.26 days in potatoes.^21^, ^22^ The half‐life of cymoxanil is influenced by factors such as pH, temperature, moisture content, and exposure to light, with degradation accelerating under alkaline conditions and higher temperatures.^23^ For example, the half‐life of cymoxanil decreases from 1.7 days at 28°C to approximately 6 h at 44 °C, showing a significant reduction in persistence as temperature increases.^23^ The half‐life decreases approximately fivefold for every 10 °C rise in temperature.^23^ Considering that the greenhouse temperature in our experiment reached a maximum of 48.3 °C, it is likely that temperature significantly contributed to the rapid degradation and short half‐life of cymoxanil in this study.

To develop a predictive model for pesticide residue dissipation in spinach, three pesticides—pencycuron, propamocarb, and cypermethrin—were selected. These pesticides were utilized to calibrate and refine the model parameters. The remaining six pesticides were reserved for validation purposes to assess the accuracy and robustness of the model predictions.

### 3.2 Model development and validation

The development of the predictive model involved adjusting plant and environmental parameters to accurately simulate pesticide residue behavior in spinach. Table 1 outlines the crop‐specific parameters utilized in the model.^24^ The plant parameters, such as organic carbon content in soil, pH, and yield, were derived from existing literature sources, while environmental parameters were obtained from experimental measurements.

Each parameter played a crucial role in shaping the model's accuracy. Soil organic carbon content affects pesticide adsorption and desorption, influencing the availability of pesticides for plant uptake.^25^ Soil pH determines the chemical stability of pesticides and can alter their degradation rates.^26^ The yield of harvested spinach impacts the dilution of pesticide residues, as higher biomass can result in lower concentrations per unit weight.

Environmental parameters, including temperature, humidity, and solar radiation, were also considered, as they significantly impact pesticide degradation pathways, including photolysis and microbial breakdown.^23^ The inclusion of these factors in the model ensures that real‐world agricultural conditions are accurately represented. Additionally, the leaf area index (LAI) was incorporated into the model to assess the role of canopy structure in pesticide deposition and dissipation.^24^

The dissipation patterns of pesticides showed a rapid decline in air and surface deposits due to volatilization, wash‐off, and photodegradation, while residues in soil and plant tissues, such as leaves, stems, and roots, exhibited slower degradation due to metabolic stability and limited translocation (Fig. 3). Systemic pesticides demonstrated gradual accumulation in stems and roots before degradation, indicating active translocation within the plant (Fig. 3). Additionally, prolonged persistence in soil suggests potential environmental accumulation, highlighting the importance of monitoring pesticide behavior across different compartments.

**Figure 2:**
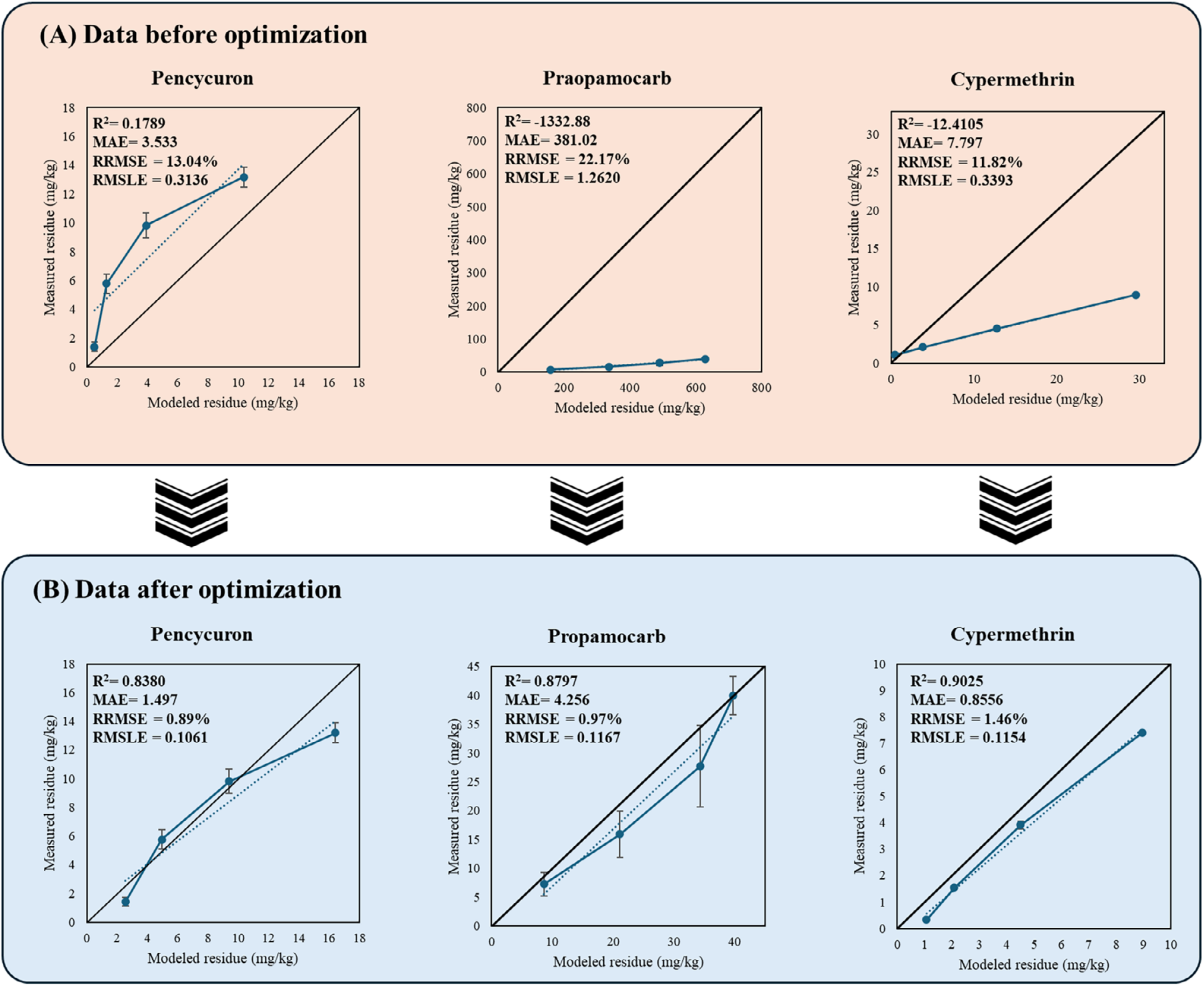
Comparison with practical data and (A) non‐optimized data, (B) optimized data.

To validate the model, the dissipation patterns of three selected pesticides (pencycuron, propamocarb, and cypermethrin) were compared with measured data before and after optimization, as illustrated in Fig. 2. Statistical evaluation of the model's performance was conducted using correlation coefficients (R^2^), MAE, RRMSE, and RMSLE. The results showed a notable improvement in model accuracy after parameter adjustments. The RRMSE values decreased from 11.82–22.17% to 0.89%–1.46%, indicating a reduction in the discrepancy between observed and predicted pesticide concentrations. In particular, the R^2^ value of propamocarb was significantly fitted from −1332.88 to 0.8797 (Fig. 2). The R^2^ values for all three pesticides exceeded 0.8, demonstrating a strong correlation between measured and modeled values. Additionally, the RRMSE values were below 2, and the RRMSE values remained under 15%, confirming the model's robustness and predictive capability.

**Figure 3:**
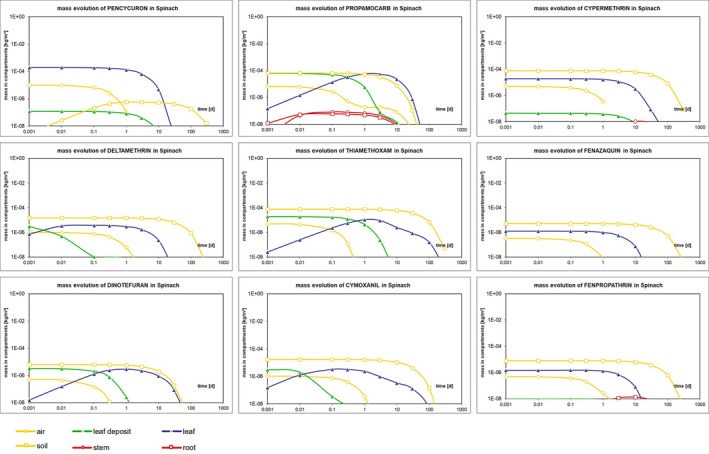
The pesticide residue mass evolution of nine pesticides in spinach ‘Leaf deposit’ represents pesticide mass retained on the leaf surface, whereas ‘leaf’ denotes pesticide mass translocated into leaf tissue. For some pesticides, stem and root residues were near‐zero and are not visually discernible.

For further validation, the developed model was applied to predict the dissipation of six additional pesticides. The comparison of predicted and observed values indicated an R^2^ range of 0.8079 to 0.9620, suggesting high model accuracy across different pesticide types. Moreover, the maximum mean absolute error (MAE) observed was 1.03 mg/kg for dinotefuran, with all pesticides maintaining an MAE below 1 mg/kg. The model is considered reliable as the MAE of the predicted pesticide residues is lower than the maximum residue limit (MRL), indicating that the prediction errors are within an acceptable range for regulatory compliance (Table 3). The RRMSE values ranged from 0.51 to 1.75, while the highest RRMSE value was recorded at 0.2179 for cymoxanil, with other pesticides staying below 0.12. These results confirm the model's reliability in predicting pesticide residues in spinach.

**Table 3:** Statistical criteria of model validation using comparison of modeled and measured values of six pesticides

Pesticide	R^2^	MAE (mg/kg)	RRMSE (%)	RMSLE
Deltamethrin	0.8079	0.13	1.41	0.1189
Thiamethoxam	0.9265	0.74	0.82	0.0983
Fenazaquin	0.9030	0.21	1.75	0.0558
Dinotefuran	0.8780	1.03	0.85	0.0664
Cymoxanil	0.8634	0.83	1.48	0.2179
Fenpropathrin	0.9620	0.26	0.51	0.0510

### 3.3 Risk assessment

To evaluate the potential health risks associated with pesticide residues, hazard quotient (HQ) values were calculated based on modeled pesticide concentrations and pre‐determined maximum residual limits (MRLs) described in Table 2. In addition to pesticides evaluated in the field trials, the modified dynamiCROP model was further applied to several commonly used pesticides in spinach cultivation to demonstrate its broader applicability for scenario‐based risk assessment. These additional simulations were conducted using crop‐specific parameters established in this study and reported physicochemical and regulatory input data. Table 4 summarizes the HQ values for six commonly used pesticides in spinach cultivation: mandipropamid, azoxystrobin, fluopicolid, cyantraniliprole, abamectin, and pymetrozine.

**Table 4:** Scenario‐based risk assessment of 15 commonly used pesticides in spinach using the modified dynamiCROP model

Pesticide	ADI (mg/kg b.w./day)	Days after the last application	Model calculatedamount (mg/kg)	EDI (mg/kg b.w./day)	HQ (%)
Pencycuron	0.053 (Japan)	14	2.56	1.1 × 10^−3^	2.13
		7	4.95	2.2 × 10^−3^	4.11
		3	9.39	4.1 × 10^−3^	7.79
		0	16.41	7.2 × 10^−3^	13.62
Propamocarb	0.29 (Europe)	14	8.63	3.8 × 10^−3^	7.17
		7	21.10	9.3 × 10^−3^	17.52
		3	34.32	1.5 × 10^−2^	28.49
		0	39.74	1.7 × 10^−2^	32.99
Cypermethrin	0.02 (Korea)	14	0.33	1.4 × 10^−4^	0.72
		7	1.54	6.8 × 10^−4^	3.39
		3	3.91	1.7 × 10^−3^	8.61
		0	7.39	3.3 × 10^−3^	16.26
Deltamethrin	0.01 (CODEX)	14	0.03	1.2 × 10^−5^	0.12
		7	0.20	9.0 × 10^−5^	0.90
		3	0.64	2.8 × 10^−4^	2.81
		0	1.22	5.4 × 10^−4^	5.38
Thiamethoxam	0.012 (USA)	14	1.31	5.7 × 10^−4^	4.79
		7	2.36	1.0 × 10^−3^	8.67
		3	4.32	1.9 × 10^−3^	15.85
		0	7.70	3.4 × 10^−3^	28.25
Fenazaquin	0.005 (Europe)	14	0.06	2.8 × 10^−5^	0.56
		7	0.30	1.3 × 10^−4^	2.60
		3	0.87	3.8 × 10^−4^	7.61
		0	1.89	8.3 × 10^−4^	16.61
Dinotefuran	0.02 (USA)	14	0.40	1.7 × 10^−4^	0.87
		7	2.17	9.6 × 10^−4^	4.78
		3	11.25	5.0 × 10^−3^	24.75
		0	12.08	5.3 × 10^−3^	26.57
Cymoxanil	0.013 (Korea)	14	0.87	3.8 × 10^−4^	2.93
		7	1.29	5.7 × 10^−4^	4.36
		3	2.27	1.0 × 10^−3^	7.70
		0	7.19	3.2 × 10^−3^	24.34
Fenpropathrin	0.03 (CODEX)	14	0.06	2.7 × 10^−5^	0.09
		7	0.17	7.4 × 10^−5^	0.25
		3	0.92	4.0 × 10^−4^	1.35
		0	3.80	1.7 × 10^−3^	5.57
Mandipropamid	0.03 (Europe)	14	0.58	2.6 × 10^−4^	0.85
		7	1.82	8.0 × 10^−4^	2.66
		3	4.81	2.1 × 10^−3^	7.06
		0	10.77	4.7 × 10^−3^	15.80
Azoxystrobin	0.18 (Japan)	14	2.18	9.6 × 10^−4^	2.13
		7	4.00	1.8 × 10^−3^	3.91
		3	8.09	3.6 × 10^−3^	7.91
		0	16.00	7.0 × 10^−3^	15.65
Fluopicolid	0.045 (Japan)	14	2.44	1.1 × 10^−3^	2.39
		7	3.46	1.5 × 10^−3^	3.38
		3	4.49	2.0 × 10^−3^	4.39
		0	5.12	2.3 × 10^−3^	5.01
Cyantraniliprole	0.057 (Korea)	14	0.92	4.1 × 10^−4^	0.71
		7	1.38	6.4 × 10^−4^	1.06
		3	1.80	7.9 × 10^−4^	1.39
		0	2.06	9.1 × 10^−4^	1.59
Abamectin	0.002 (Korea)	14	0.01	3.4 × 10^−6^	0.01
		7	0.02	8.8 × 10^−6^	0.02
		3	0.05	2.1 × 10^−5^	0.04
		0	0.09	4.0 × 10^−5^	0.07
Pymetrozin	0.0038 (USA)	14	0.03	1.3 × 10^−5^	0.33
		7	0.24	1.1 × 10^−4^	2.81
		3	1.00	4.4 × 10^−4^	11.52
		0	2.61	1.2 × 10^−3^	30.28

Among these pesticides, propamocarb exhibited the highest HQ value of 32.99% immediately after application (Day 0) (Table 4). Generally, an HQ value below 100% is considered to pose negligible risk; however, since spinach is rarely consumed alone in large quantities, an HQ exceeding 30% may still indicate potential health concerns.^27^ Unlike other pesticides, the data suggested that spinach cultivated by spraying propamocarb should not be harvested immediately.

Spinach is one of the most widely consumed leafy vegetables globally, forming a key component of various diets due to its high nutritional value. The high intake of spinach in numerous cultures makes it essential to rigorously assess pesticide residue levels to prevent potential health risks. Given the increased consumption rates, the need for reliable risk assessment models becomes even more critical.

A substantial temporal decline in HQ values was observed, with all pesticides falling below 10% by Day 14 post‐application (Table 4). This finding implies that a minimum waiting period of 14 days after pesticide application is necessary to ensure safe consumption of spinach. Ensuring proper pre‐harvest intervals based on modeled predictions can help mitigate health risks associated with pesticide exposure.

The importance of risk assessment is further underscored by global trends in pesticide regulations, which increasingly demand stricter safety evaluations before approving pesticide applications.^28^ Many regulatory bodies, including the European Food Safety Authority (EFSA) and the United States Environmental Protection Agency (EPA), mandate extensive risk assessments to establish maximum residue limits (MRLs). Predictive modeling, as presented in this study, offers an efficient tool for aligning agricultural practices with these regulatory requirements while minimizing unnecessary pesticide applications.

These results highlight the importance of using predictive models to assess pesticide exposure risks and optimize application schedules. By integrating these models into agricultural practices, farmers can make informed decisions on pesticide use while safeguarding consumer health. Additionally, regulatory agencies can utilize such models to establish evidence‐based maximum residue limits, promoting food safety on a larger scale. The ability to predict potential risks before pesticide application also contributes to sustainable agriculture, ensuring a balance between effective pest control and human health protection.

## 4 CONCLUSION

This study adapted and validated the dynamiCROP model for spinach, a leafy vegetable not previously included in its standard framework. Through the incorporation of spinach‐specific physiological parameters and localized environmental data, the model demonstrated high predictive accuracy in simulating the dissipation dynamics of nine commonly used pesticides. The use of multi‐year field data under varying environmental conditions strengthened the robustness of model calibration, as dynamiCROP explicitly incorporates temperature and humidity as dynamic inputs rather than assuming constant conditions.

Rather than proposing a novel modeling framework, this research offers a practical extension of an established tool to a high‐risk and widely consumed crop. The findings confirm that model‐predicted pesticide concentrations, when integrated into hazard quotient (HQ) calculations, can effectively support dietary risk assessment. Notably, all tested compounds exhibited HQ values below 10% by 14 days post‐application, supporting the implementation of a minimum pre‐harvest interval for consumer safety. This approach illustrates how the calibrated model can be used as a predictive tool to estimate potential dietary risks of pesticides that were not directly tested in field experiments, supporting proactive risk management.

By improving the resolution of predictive modeling for pesticide residues in spinach, this study contributes to the refinement of crop‐specific residue management. The adapted model can aid in optimizing application strategies, informing regulatory limits, and enhancing food safety protocols. While the core algorithm remains unchanged, this study highlights the importance of parameter localization and field validation in extending the usability of pesticide fate models in crops that lack representation but are highly contaminated with pesticides, such as spinach.

This study contributes practical insights into risk‐based pesticide management, supporting food safety regulators and agricultural stakeholders.

## CONFLICT OF INTEREST

The authors declare the following financial interests/personal relationships which may be considered as potential competing interests.

## AUTHOR CONTRIBUTIONS

Ji‐Woo Yu: Writing – original draft, Software, Visualization, Validation, Methodology, Investigation. Min‐Ho Song: Investigation, Methodology, Software. Jung‐Hoon Lee: Data curation, Formal analysis. Hui‐Yeon Ahn: Data curation, Formal analysis. Eun‐Song Choi: Data curation, Formal analysis. Young‐Soo Keum: Resources. Hyun Ho Noh: Conceptualization, Project administration. Ji‐Ho Lee: Conceptualization, Funding acquisition, Project administration, Supervision, Writing‐review & editing.

## Supporting information


**Table S1.** Field test duration.
**Table S2.** Temperature and humidity in greenhouse environments during pesticide application.
**Table S3.** Physical properties of nine pesticides.
**Table S4.** Analytical conditions for pesticide quantitation.
**Table S5.** Method validation results for nine pesticides.
**Table S6.** Residual pesticide in spinach.

## Data Availability

The data that support the findings of this study are available on request from the corresponding author. The data are not publicly available due to privacy or ethical restrictions.
