# Supplementary material for: Integrating DynamiCROP model and risk assessment for pesticide residues in spinach: Implications for food safety
Source: Pest Manag Sci. 2026 Apr 12;82(7):7154–63. doi: 10.1002/ps.70799 (PMC13240685; doi:10.1002/ps.70799)
Supplement: Supplementary file 1 — Table S1. Field test duration. Table S2. Temperature and humidity in greenhouse environments during pesticide application. Table S3. Physical properties of nine pesticides. Table S4. Analytical conditions for pesticide quantitation. Table S5. Method validation results for nine pesticides. Table S6. Residual pesticide in spinach. [file PS-82-7154-s001.docx]

**Title: Integrating DynamiCROP Model and Risk Assessment for Pesticide Residues in Spinach: Implications for Food Safety**

Ji-Woo Yu^1†^, Min-Ho Song^2†^, Jung-Hoon Lee^2,3^, Hui-Yeon Ahn^1^, Eun-Song Choi^2,3^, Youn-Soo Keum^1^, Hyun Ho Noh^4*^, and Ji-Ho Lee^2,3*^

*^1^Department of Crop Science, College of Sang-Huh Life Science, Konkuk University, Seoul, Republic of Korea*

*^2^School of Natural Resources and Environment Science, College of Agriculture and Life Sciences, Kangwon National University, Gangwon State, Republic of Korea*

*^3^Department of Food Biotechnology and Environmental Science, Kangwon National University, Chuncheon, Gangwon State, 24341, Kore*

*^4^Residual Agrochemical Assessment Division, National Institute of Agricultural Sciences, Wanju, Republic of Korea*

*Correspondence

*Corresponding author:

Ji-Ho Lee, micai@kangwon.ac.kr

Hyun Ho Noh, noh1983@korea.kr

^†^ These authors contributed equally

Table S1. Field test duration.

| **Duration** | **Tested pesticides** |
| --- | --- |
| 2016-04-12 to 2016-06-09 | Pencyrcuron, propamocarb |
| 2017-02-07 to 2017-04-04 | Cypermethrin, deltamethrin, thiamethoxam |
| 2018-02-09 to 2018-04-06 | Fenazaquin |
| 2021-02-18 to 2021-04-15 | Dinotefuran |
| 2022-02-28 to 2022-04-28 | Cymoxanil, fenpropathrin |

Table S2. Temperature and humidity in greenhouse environments during pesticide application.

| **Date** | **Temperature**  **(average, ℃)** | **Humidity**  **(average, %)** | **Pesticide application** |
| --- | --- | --- | --- |
| 2016-05-12 | 21.5 | 29.4 | Pesticide application 28 days before harvest |
| 2016-05-13 | 18.7 | 30.6 |  |
| 2016-05-14 | 20.7 | 39.3 |  |
| 2016-05-15 | 21.3 | 31.9 |  |
| 2016-05-16 | 18 | 27.7 |  |
| 2016-05-17 | 21.1 | 42.6 |  |
| 2016-05-18 | 24.6 | 46.6 |  |
| 2016-05-19 | 24.5 | 48.4 | Pesticide application 21 days before harvest |
| 2016-05-20 | 25.2 | 47.1 |  |
| 2016-05-21 | 22.4 | 47.6 |  |
| 2016-05-22 | 23.3 | 44.6 |  |
| 2016-05-23 | 24.9 | 43.3 | Pesticide application 17 days before harvest |
| 2016-05-24 | 20.4 | 28.6 |  |
| 2016-05-25 | 25.4 | 45.1 |  |
| 2016-05-26 | 25.1 | 43.1 | Pesticide application 14 days before harvest |
| 2016-05-27 | 27.7 | 46.8 |  |
| 2016-05-28 | 26.9 | 45.4 |  |
| 2016-05-29 | 24.9 | 44.3 |  |
| 2016-05-30 | 27.2 | 47.9 | Pesticide application 10 days before harvest |
| 2016-05-31 | 25.9 | 43.1 |  |
| 2016-06-01 | 24.6 | 37.4 |  |
| 2016-06-02 | 25.7 | 45.4 | Pesticide application 7 days before harvest |
| 2016-06-03 | 26.3 | 45.4 |  |
| 2016-06-04 | 20.7 | 25.5 |  |
| 2016-06-05 | 23.9 | 38.3 |  |
| 2016-06-06 | 18.8 | 21.4 | Pesticide application 3 days before harvest |
| 2016-06-07 | 26.5 | 46.4 |  |
| 2016-06-08 | 23 | 31.5 |  |
| 2016-06-09 | 28.2 | 47.4 | Pesticide application 0 days before harvest |

Table S2. *Continued*.

| **Date** | **Temperature**  **(average, ℃)** | **Humidity**  **(average, %)** | **Pesticide application** |
| --- | --- | --- | --- |
| 2017-03-07 | 13.3 | 78.3 | Pesticide application 28 days before harvest |
| 2017-03-08 | 12.4 | 76.1 |  |
| 2017-03-09 | 11.8 | 73.2 |  |
| 2017-03-10 | 12.3 | 69.6 |  |
| 2017-03-11 | 12.8 | 68.6 |  |
| 2017-03-12 | 14.5 | 66.6 |  |
| 2017-03-13 | 14.7 | 65.9 |  |
| 2017-03-14 | 13.2 | 63.5 | Pesticide application 21 days before harvest |
| 2017-03-15 | 11.5 | 61.1 |  |
| 2017-03-16 | 13.8 | 65.8 |  |
| 2017-03-17 | 14.6 | 69.7 |  |
| 2017-03-18 | 11.9 | 70.5 | Pesticide application 17 days before harvest |
| 2017-03-19 | 13.4 | 66.6 |  |
| 2017-03-20 | 9.9 | 74 |  |
| 2017-03-21 | 11.8 | 66.4 | Pesticide application 14 days before harvest |
| 2017-03-22 | 11 | 62.3 |  |
| 2017-03-23 | 13.2 | 52.9 |  |
| 2017-03-24 | 11.8 | 59.4 |  |
| 2017-03-25 | 9.5 | 83.4 | Pesticide application 10 days before harvest |
| 2017-03-26 | 9.6 | 74 |  |
| 2017-03-27 | 6.9 | 68.1 |  |
| 2017-03-28 | 10.7 | 56.6 | Pesticide application 7 days before harvest |
| 2017-03-29 | 12.5 | 57.8 |  |
| 2017-03-30 | 14.5 | 49.6 |  |
| 2017-03-31 | 10.8 | 53.5 |  |
| 2017-04-01 | 10.2 | 68.7 | Pesticide application 3 days before harvest |
| 2017-04-02 | 11.7 | 52.5 |  |
| 2017-04-03 | 12.3 | 43.4 |  |
| 2017-04-04 | 16.4 | 37.2 | Pesticide application 0 days before harvest |

Table S2. *Continued*.

| **Date** | **Temperature**  **(average, ℃)** | **Humidity**  **(average, %)** | **Pesticide application** |
| --- | --- | --- | --- |
| 2018-03-09 | 15.2 | 27.8 | Pesticide application 28 days before harvest |
| 2018-03-10 | 11.2 | 29.8 |  |
| 2018-03-11 | 12.5 | 31.6 |  |
| 2018-03-12 | 13.9 | 32.5 |  |
| 2018-03-13 | 15.5 | 32.8 |  |
| 2018-03-14 | 18 | 37.7 |  |
| 2018-03-15 | 16.3 | 21.4 |  |
| 2018-03-16 | 10.2 | 18.1 | Pesticide application 21 days before harvest |
| 2018-03-17 | 11.1 | 31 |  |
| 2018-03-18 | 11.8 | 27.6 |  |
| 2018-03-19 | 9.7 | 12.3 |  |
| 2018-03-20 | 7.7 | 15.7 | Pesticide application 17 days before harvest |
| 2018-03-21 | 2.8 | 5.8 |  |
| 2018-03-22 | 10.3 | 26.6 |  |
| 2018-03-23 | 10.6 | 30 | Pesticide application 14 days before harvest |
| 2018-03-24 | 12.5 | 29.5 |  |
| 2018-03-25 | 14.8 | 35.3 |  |
| 2018-03-26 | 14.8 | 33.1 |  |
| 2018-03-27 | 16.6 | 34.5 | Pesticide application 10 days before harvest |
| 2018-03-28 | 18.4 | 38.9 |  |
| 2018-03-29 | 17.2 | 30.3 |  |
| 2018-03-30 | 15.9 | 28.4 | Pesticide application 7 days before harvest |
| 2018-03-31 | 16.5 | 34.4 |  |
| 2018-04-01 | 17.5 | 31.3 |  |
| 2018-04-02 | 19.9 | 34.8 |  |
| 2018-04-03 | 21.1 | 37.6 | Pesticide application 3 days before harvest |
| 2018-04-04 | 12.3 | 15.8 |  |
| 2018-04-05 | 9.2 | 11.3 |  |
| 2018-04-06 | 14.5 | 25.6 | Pesticide application 0 days before harvest |

Table S2. *Continued*.

| **Date** | **Temperature**  **(average, ℃)** | **Humidity**  **(average, %)** | **Pesticide application** |
| --- | --- | --- | --- |
| 2021-03-18 | 19.2 | 37.5 | Pesticide application 28 days before harvest |
| 2021-03-19 | 16.9 | 30.7 |  |
| 2021-03-20 | 12.4 | 17.9 |  |
| 2021-03-21 | 18 | 34 |  |
| 2021-03-22 | 16.8 | 35.2 |  |
| 2021-03-23 | 17.3 | 42.8 |  |
| 2021-03-24 | 14.9 | 35 |  |
| 2021-03-25 | 21.1 | 46.6 | Pesticide application 21 days before harvest |
| 2021-03-26 | 19.6 | 39 |  |
| 2021-03-27 | 12.7 | 20.8 |  |
| 2021-03-28 | 20.6 | 37.9 |  |
| 2021-03-29 | 17.3 | 34.3 | Pesticide application 17 days before harvest |
| 2021-03-30 | 20.4 | 44.1 |  |
| 2021-03-31 | 19.8 | 41.8 |  |
| 2021-04-01 | 17.3 | 32.1 | Pesticide application 14 days before harvest |
| 2021-04-02 | 21.7 | 39.1 |  |
| 2021-04-03 | 18.2 | 28.8 |  |
| 2021-04-04 | 14.3 | 18.8 |  |
| 2021-04-05 | 18.9 | 43.1 | Pesticide application 10 days before harvest |
| 2021-04-06 | 19.1 | 39.4 |  |
| 2021-04-07 | 21.6 | 46.6 |  |
| 2021-04-08 | 22.1 | 44.2 | Pesticide application 7 days before harvest |
| 2021-04-09 | 15.3 | 31.3 |  |
| 2021-04-10 | 18.2 | 38.1 |  |
| 2021-04-11 | 20.2 | 41.5 |  |
| 2021-04-12 | 13.7 | 19.5 | Pesticide application 3 days before harvest |
| 2021-04-13 | 21.4 | 42 |  |
| 2021-04-14 | 18.1 | 39.4 |  |
| 2021-04-15 | 15.5 | 30.2 | Pesticide application 0 days before harvest |

Table S2. *Continued*.

| **Date** | **Temperature**  **(average, ℃)** | **Humidity**  **(average, %)** | **Pesticide application** |
| --- | --- | --- | --- |
| 2022-03-31 | 16.8 | 29.1 | Pesticide application 28 days before harvest |
| 2022-04-01 | 17.3 | 39 |  |
| 2022-04-02 | 17.4 | 40.4 |  |
| 2022-04-03 | 18.3 | 42.1 |  |
| 2022-04-04 | 18.9 | 41.7 |  |
| 2022-04-05 | 16.9 | 33.5 |  |
| 2022-04-06 | 19.9 | 42.8 |  |
| 2022-04-07 | 16.7 | 33.1 | Pesticide application 21 days before harvest |
| 2022-04-08 | 18.6 | 38.5 |  |
| 2022-04-09 | 20 | 39.4 |  |
| 2022-04-10 | 20.7 | 38.8 |  |
| 2022-04-11 | 25.2 | 46.7 | Pesticide application 17 days before harvest |
| 2022-04-12 | 26.7 | 48.3 |  |
| 2022-04-13 | 15.7 | 19.7 |  |
| 2022-04-14 | 13.8 | 22.3 | Pesticide application 14 days before harvest |
| 2022-04-15 | 17.8 | 34.9 |  |
| 2022-04-16 | 19.2 | 38 |  |
| 2022-04-17 | 17.4 | 33.2 |  |
| 2022-04-18 | 19.3 | 36.5 | Pesticide application 10 days before harvest |
| 2022-04-19 | 20.9 | 40.4 |  |
| 2022-04-20 | 21.3 | 41.8 |  |
| 2022-04-21 | 15.3 | 30.1 | Pesticide application 7 days before harvest |
| 2022-04-22 | 20.3 | 40.2 |  |
| 2022-04-23 | 23.8 | 40 |  |
| 2022-04-24 | 25.2 | 41.8 |  |
| 2022-04-25 | 24.7 | 41.5 | Pesticide application 3 days before harvest |
| 2022-04-26 | 20 | 27.8 |  |
| 2022-04-27 | 23 | 39.4 |  |
| 2022-04-28 | 21.7 | 37.1 | Pesticide application 0 days before harvest |

Table S3. Physical properties of nine pesticides

| **Pesticide** | **Molecular formula** | **Chemical group** | **Water solubility** | **Vapor pressure** | **Melting point** |
| --- | --- | --- | --- | --- | --- |
| **Cypermethrin** | C_22_H_19_Cl_2_NO_3_ | Pyrethroid | 3.97 μg/L  (20℃) | 6.9×10^-2^  Pa m^3^ mol^-1^ | 81.5℃ |
| **Deltamethrin** | C_22_H_19_Br_2_NO_3_ | Pyrethroid | < 0.2 μg/L  (25℃) | 3.13×10^-2^  Pa m^3^ mol^-1^ | 100-102℃ |
| **Fenpropathrin** | C_22_H_23_NO_3_ | Pyrethroid | 14.1 μg/L  (25℃) | 0.73  mPa | 45-50℃ |
| **Thiamethoxam** | C_8_H_10_ClN_5_O_3_S | Neonicotinoid | 4.1 g/L  (25℃) | 4.70×10^-10^  Pa m^3^ mol^-1^ | 139.1℃ |
| **Dinotefuran** | C_7_H_14_N_4_O_3_ | Neonicotinoid | 39.8 g/L  (20℃) | 8.4×10^-9^  Pa m^3^ mol^-1^ | 107.5℃ |
| **Pencycuron** | C_19_H_21_ClN_2_O | Phenylurea fungicide | 0.3 mg/L (20℃) | 5×10^-7^  Pa m^3^ mol^-1^ | 128℃ |
| **Propamocarb** | C_9_H_20_N_2_O_2_ | Carbamate fungicide | >500 g/L  (20℃) | 8.5×10^-9^  Pa m^3^ mol^-1^ | 64.2℃ |
| **Fenazaquin** | C_20_H_22_N_2_O | Mitochondrial complex I electron transport inhibitors  (METI) | 0.22 mg/L  (20℃) | 3.4×10^-3^  Pa m^3^ mol^-1^ | 77.5-80.0℃ |
| **Cymoxanil** | C_7_H_10_N_4_O_3_ | Cyanoacetamide oxime | 0.89 g/L  (20℃) | 3.8×10^-5^  Pa m^3^ mol^-1^ | 160℃ |

Table S4. Analytical conditions for pesticide quantitation

| **Pencycuron** | |
| --- | --- |
| **Instrument** | Waters e2695 |
| **Detector** | UV detector (Waters 2489, wavelength : 240 nm) |
| **Column** | XDB-C18 (5μm, 4.6 x 250 mm) |
| **Mobile phase** | Acetonitrile/Water (70/30, v/v) |
| **Column oven** | 25℃ |
| **Flow rate** | 1.0 mL/min |
| **Injection volume** | 20.0 μL |
| **Retention time** | 5.6 min |

| **Propamocarb** | |
| --- | --- |
| **Instrument** | Agilent Technologies 7890B |
| **Detector** | NPD |
| **Column** | DB-WAX(30m, 0.25 μm film thickness, 0.32 mm I.d.; Agilent, USA) |
| **Column oven** | 50℃(0.5 min) → 20℃/min → 220℃(0 min) |
| **Detector temperature** | 280℃ |
| **Injection temperature** | 250℃ |
| **Injection mode** | Split (10:1) |
| **Carrier gas (N_2_)** | 2.0 mL/min |
| **Make up gas (N_2_)** | 10.0 mL/min |
| **Detector gas (air)** | 60.0 mL/min |
| **Detector gas (H_2_)** | 3.0 mL/min |
| **Injection volume** | 2.0 μL |
| **Retention time** | 9.09 min |

| **Cypermethrin** | |
| --- | --- |
| **Instrument** | Agilent Technologies 7890B |
| **Detector** | ECD |
| **Column** | DB-17(30m, 0.25 μm film thickness, 0.32 mm I.d.; Agilent, USA) |
| **Column oven** | 50℃(0.5 min) → 20℃/min → 220℃(0 min) |
| **Detector temperature** | 280℃ |
| **Injection temperature** | 260℃ |
| **Injection mode** | Splitless |
| **Carrier gas (N_2_)** | 2.0 mL/min |
| **Make up gas (N_2_)** | 15.0 mL/min |
| **Detector gas (air)** | 60.0 mL/min |
| **Detector gas (H_2_)** | 3.0 mL/min |
| **Injection volume** | 1.0 μL |
| **Retention time** | 14.6 min |

| **Deltamethrin** | |
| --- | --- |
| **Instrument** | Agilent Technologies 7890B |
| **Detector** | ECD |
| **Column** | HP-5(30m, 0.25 μm film thickness, 0.32 mm I.d.; Agilent, USA) |
| **Column oven** | 250℃(10 min) → 1℃/min → 260℃(10 min) |
| **Detector temperature** | 280℃ |
| **Injection temperature** | 260℃ |
| **Injection mode** | Splitless |
| **Carrier gas (N_2_)** | 2.0 mL/min |
| **Make up gas (N_2_)** | 15.0 mL/min |
| **Detector gas (air)** | 60.0 mL/min |
| **Detector gas (H_2_)** | 3.0 mL/min |
| **Injection volume** | 1.0 μL |
| **Retention time** | 13.6 min |

| **Thiamethoxam** | |
| --- | --- |
| **Instrument** | Waters e2695 |
| **Detector** | UV detector (Waters 2489, wavelength : 240 nm) |
| **Column** | Phenomenex Luna C18(2) 100 Å(5μm, 250 x 4.6 mm) |
| **Mobile phase** | Acetonitrile/0.1% Formic acid in Water : 15/85(0min) → 40/60(9min) → 90/10(13min) → 15/85(13.1min) → 15/85(18min) |
| **Column oven** | 25℃ |
| **Flow rate** | 1.0 mL/min |
| **Injection volume** | 20.0 μL |
| **Retention time** | 8.5 min |

| **Deltamethrin** | |
| --- | --- |
| **Instrument** | Agilent Technologies 7890B |
| **Detector** | NPD |
| **Column** | DB-17 (30m, 0.25 μm film thickness, 0.32 mm I.d.; Agilent, USA) |
| **Column oven** | 80℃(2 min) → 10℃/min → 280℃(8 min) |
| **Detector temperature** | 280℃ |
| **Injection temperature** | 260℃ |
| **Injection mode** | Splitless |
| **Carrier gas (N_2_)** | 2.0 mL/min |
| **Make up gas (N_2_)** | 5.0 mL/min |
| **Detector gas (air)** | 120.0 mL/min |
| **Detector gas (H_2_)** | 3.0 mL/min |
| **Injection volume** | 1.0 μL |
| **Retention time** | 23.8 min |

| **Dinotefuran** | |
| --- | --- |
| **Instrument** | AB Sciex Exion LC (Japan) |
| **Detector** | Tandem Mass spectrometer API 3200 (Japan) |
| **Column** | Luna® 5μm C18(2) 100 Å(Phenomenex Co., USA), (250 mm x 4.6 mm i.d.) |
| **Mobile phase** | A - 0.1% formic acid + 5 mM ammonium formate in Methanol  B - 0.1% formic acid + 5 mM ammonium formate in Water |
| **Flow rate** | 1 mL/min |
| **Column oven** | 40℃ |
| **Injection volume** | 10 μL |
| **Ionization mode** | Electrospray ionization (ESI) |
| **Ionspray voltage** | 4500 V |
| **Interface temperature** | 300℃ |
| **Ion source gas** | 30 psi |
| **Curtain gas** | 30 psi |
| **Collision gas** | 10 psi |

| **Cymoxanil** | |
| --- | --- |
| **Instrument** | AB Sciex Exion LC (Japan) |
| **Detector** | Tandem Mass spectrometer API 3200 (Japan) |
| **Column** | Acquity UPLC® BEH Shield RP18 1.7μm (2.1 x 100 mm Column) |
| **Mobile phase** | A - 0.1% formic acid + 5 mM ammonium formate in Water  B - 0.1% formic acid + 5 mM ammonium formate in Acetonitrile |
| **Flow rate** | 0.15 mL/min |
| **Column oven** | 40℃ |
| **Injection volume** | 2 μL |
| **Ionization mode** | Electrospray ionization (ESI) |
| **Ionspray voltage** | 4500 V |
| **Interface temperature** | 400℃ |
| **Ion source gas** | 50 psi |
| **Curtain gas** | 20 psi |
| **Collision gas** | 5 psi |
| **Cymoxanil** | |
| **Instrument** | Shimadzu GC-MS TQ8050 NX(Japan) |
| **Detector** | Tandem Mass Detector |
| **Column** | Rxi-5ms (Restek, USA) (30m x 0.25, 0.50μm) |
| **Carrier gas** | He |
| **Flow rate** | 2 mL/min |
| **Column oven** | 40℃ |
| **Injection volume** | 2 μL |
| **Ionization mode** | Electron ionization (EI) |
| **Ion source temperature** | 230℃ |
| **Interface temperature** | 280℃ |
| **Injection mode** | Splitless |
| **Detector voltage** | 1.5 kV |
| **Colum flow** | 1.03 mL/min |

Table S5. Method validation results for nine pesticides

| **Pesticide** | **LOQ^*^**  **(mg/kg)** | **Calibration curve** | **R^2^** | **Recovery**  **level**  **(mg/kg)** | **Recovery** $\boldsymbol{\pm}$ **RSD^**^ (%)** |
| --- | --- | --- | --- | --- | --- |
| **Pencycuron** | 0.3 | y=3814.6x+6683.4 | 1.0000 | 0.3 | 100.3 ± 6.0 |
|  |  |  |  | 1.6 | 101.7 ± 2.2 |
| **Propamocarb** | 0.05 | y=24.971x+-0.0457 | 0.9992 | 0.05 | 96.8 ± 5.1 |
|  |  |  |  | 0.25 | 87.6 ± 3.8 |
| **Cypermethrin** | 0.1 | y=149,503.9740x+0.9980 | 0.9980 | 0.1 | 106.8 ± 14.1 |
|  |  |  |  | 0.5 | 113.7 ± 0.8 |
|  |  |  |  | 20 | 94.8 ± 3.5 |
| **Deltamethrin** | 0.1 | y=213,242.0970x+3,477.6962 | 0.9993 | 0.1 | 84.9 ± 10.4 |
|  |  |  |  | 0.5 | 98.4 ± 15.9 |
| **Thiamethoxam** | 0.4 | y=2,074.4887x-52.4895 | 1.0000 | 0.4 | 77.5 ± 8.1 |
|  |  |  |  | 2.0 | 76.2 ± 3.6 |
|  |  |  |  | 10 | 76.3 ± 6.1 |
| **Fenazaquin** | 0.5 | y=48.9625x+0.1199 | 1.0000 | 0.5 | 108.6 ± 9.3 |
|  |  |  |  | 2.5 | 102.8 ± 3.5 |
| **Dinotefuran** | 0.02 | y=83,361.9420x+1,079.1702 | 0.9996 | 0.02 | 78.8 ± 5.6 |
|  |  |  |  | 0.2 | 89.6 ± 1.8 |
|  |  |  |  | 12 | 95.7 ± 2.2 |
| **Cymoxanil** | 0.01 | y=211558.3401x+1442.6326 | 0.9959 | 0.01 | 79.6 ± 12.5 |
|  |  |  |  | 0.1 | 105.0 ± 5.9 |
|  |  |  |  | 7.0 | 90.5 ± 1.3 |
| **Fenpropathrin** | 0.01 | y=168894.3534x+421.8958 | 0.9953 | 0.01 | 86.9 ± 3.1 |
|  |  |  |  | 0.1 | 90.3 ± 10.2 |
|  |  |  |  | 5.0 | 77.7 ± 7.1 |

^*^ LOQ: limit of quantitation, ^**^ RSD: relative standard deviation.

**Table S6. Residual pesticide in spinach**

| **Pesticide** | **Application amount (active ingredient kg/10a)** | **Application date**  **(days before harvest)** ^*^ | **Residual concentration**  **(mg/kg, 3 replicates)** | | | **Average ± SD**^**^ |
| --- | --- | --- | --- | --- | --- | --- |
| **Pencycuron** | 0.0002 kg/10a | 28-21-14 | 1.25 | 1.76 | 1.22 | 1.41 ± 0.30 |
|  |  | 21-14-7 | 6.36 | 5.02 | 5.93 | 5.77 ± 0.68 |
|  |  | 17-10-3 | 8.85 | 10.15 | 10.47 | 9.82 ± 0.86 |
|  |  | 14-7-0 | 12.39 | 13.68 | 13.48 | 13.18 ± 0.69 |
| **Propamocarb** | 0.00133 kg/10a | 28-21-14 | 5.83 | 6.43 | 9.61 | 7.29 ± 2.03 |
|  |  | 21-14-7 | 18.05 | 18.44 | 11.23 | 15.91 ± 4.05 |
|  |  | 17-10-3 | 21.72 | 35.47 | 25.87 | 27.69 ± 7.05 |
|  |  | 14-7-0 | 43.68 | 37.43 | 38.68 | 39.93 ± 3.31 |
| **Cypermethrin** | 0.0001 kg/10a | 21-14 | 1.18 | 1.08 | 0.92 | 1.06 ± 0.13 |
|  |  | 14-7 | 2.1 | 1.98 | 2.15 | 2.08 ± 0.09 |
|  |  | 10-3 | 4.25 | 5.01 | 4.26 | 4.51 ± 0.44 |
|  |  | 7-0 | 8.37 | 9.92 | 8.55 | 8.95 ± 0.85 |
| **Deltamethrin** | 0.000002 kg/10a | 21-14 | 0.09 | 0.06 | 0.09 | 0.08 ± 0.02 |
|  |  | 14-7 | 0.38 | 0.29 | 0.27 | 0.31 ± 0.06 |
|  |  | 10-3 | 0.9 | 0.61 | 0.66 | 0.72 ± 0.16 |
|  |  | 7-0 | 0.92 | 0.99 | 0.97 | 0.96 ± 0.04 |
| **Thiamethoxam** | 0.00001 kg/10a | 21-14 | 0.64 | 0.71 | 0.54 | 0.63 ± 0.09 |
|  |  | 14-7 | 1.75 | 3.11 | 3.04 | 2.63 ± 0.77 |
|  |  | 10-3 | 4.56 | 5.85 | 5.44 | 5.28 ± 0.66 |
|  |  | 7-0 | 7.09 | 7.81 | 6.44 | 7.11 ± 0.69 |
| **Fenazaquin** | 0.0000067 kg/10a | 28-21-14 | 0.16 | 0.19 | 0.17 | 0.17 ± 0.02 |
|  |  | 21-14-7 | 0.46 | 0.36 | 0.32 | 0.38 ± 0.07 |
|  |  | 17-10-3 | 1.37 | 1.27 | 1.15 | 1.26 ± 0.11 |
|  |  | 14-7-0 | 1.95 | 1.93 | 2.48 | 2.12 ± 0.31 |
| **Dinotefuran** | 0.00001 kg/10a | 21-14 | 0.49 | 0.61 | 0.51 | 0.54 ± 0.06 |
|  |  | 14-7 | 2.44 | 2 | 2.29 | 2.24 ± 0.22 |
|  |  | 10-3 | 8.28 | 8.04 | 8.78 | 8.37 ± 0.38 |
|  |  | 7-0 | 11.56 | 11.24 | 10.66 | 11.15 ± 0.46 |
| **Cymoxanil** | 0.000021 kg/10a | 21-14 | 0.01 | 0.01 | 0.01 | 0.01 ± 0.00 |
|  |  | 14-7 | 0.05 | 0.04 | 0.06 | 0.05 ± 0.01 |
|  |  | 10-3 | 2.21 | 2.1 | 2.5 | 2.27 ± 0.21 |
|  |  | 7-0 | 6.06 | 6.54 | 5.79 | 6.13 ± 0.38 |
| **Fenpropathrin** | 0.00001 kg/10a | 21-14 | 0.03 | 0.03 | 0.02 | 0.03 ± 0.01 |
|  |  | 14-7 | 0.36 | 0.32 | 0.36 | 0.35 ± 0.02 |
|  |  | 10-3 | 1.21 | 1.17 | 1.35 | 1.24 ± 0.09 |
|  |  | 7-0 | 3.7 | 3.26 | 3.35 | 3.44 ± 0.23 |

^*^ Values such as “28-21-14” indicate multiple application timings conducted 28, 21, and 14 days before harvest.

^**^ SD: standard deviation.
